# Electro-acupuncture attenuates inflammatory responses and intraabdominal pressure in septic patients: A randomized controlled trial

**DOI:** 10.1097/MD.0000000000010555

**Published:** 2018-04-27

**Authors:** Jian-biao Meng, Yan-na Jiao, Xiu-juan Xu, Zhi-zhen Lai, Geng Zhang, Chun-lian Ji, Ma-hong Hu

**Affiliations:** a Intensive Care Unit, Tongde Hospital of Zhejiang Province; b Intensive Care Unit, The First Affiliated Hospital, College of Medicine, Zhejiang University, Hangzhou, China.

**Keywords:** electro-acupuncture, inflammatory response, intestinal dysfunction, intraabdominal pressure, sepsis, syndrome of obstruction of the bowels Qi

## Abstract

**Background:**

A pathological increase in intraabdominal pressure (IAP) and inflammatory responses have negative effects on splanchnic, respiratory, cardiovascular, renal, and neurological function in septic patients with intestinal dysfunction. Electro-acupuncture (EA) has been evidenced to have a bidirectional neuron-endocrine-immune system regulating effect in patients with intestinal dysfunction. The purpose of current study was to evaluate the effects of EA at “Zusanli” (ST36) and “Shangjuxu” (ST37) on inflammatory responses and IAP in septic patients with intestinal dysfunction manifested syndrome of obstruction of the bowels Qi.

**Methods:**

Eighty-two septic patients with intestinal dysfunction manifested syndrome of obstruction of the bowels Qi were randomly assigned to control group (n = 41) and EA group (n = 41). Patients in control group were given conventional therapies including fluid resuscitation, antiinfection, vasoactive agents, mechanical ventilation (MV), supply of enteral nutrition, and glutamine as soon as possible. In addition to conventional therapies, patients in EA group underwent 20-minutes of EA at ST36-ST37 twice a day for 5 days. At baseline, posttreatment 1, 3, and 7 days, serum levels of tumor necrosis factor-α (TNF-α) and interleukin-1β (IL-1β) and IAP levels, were measured, respectively. And days on MV, length of stay in intensive care unit (ICU) and 28 days mortality were recorded.

**Results:**

The serum levels of TNF-α and IL-1β and IAP levels at posttreatment 1, 3, and 7 days were lower significantly in the EA group compared with the control group (mean [SD]; 61.03 [20.39] vs 79.28 [20.69]; *P* < .005, mean [SD]; 35.34 [18.75] vs 66.53 [30.43]; *P* < .005 and mean [SD]; 20.32 [11.30] vs 32.99 [20.62]; *P* = .001, respectively, TNF-α. Mean [SD]; 14.11 [5.21] vs 16.72 [5.59]; *P* = .032, mean [SD]; 9.02 [3.62] vs 12.10 [4.13]; *P* = .001 and mean [SD]; 5.11 [1.79] vs 8.19 [2.99]; *P* < .005, respectively, IL-1β. Mean [SD]; 14.83 [5.58] vs 17.55 [3.37]; *P* = .009, mean [SD]; 11.20 [2.57] vs 14.85 [3.01]; *P* < .005 and mean [SD]; 8.62 [2.55] vs 11.25 [2.72]; *P* < .005, respectively, IAP). There were no significant differences in the duration of MV, length of stay in ICU, and 28d mortality between the groups.

**Conclusion:**

EA at ST36-ST37 attenuated inflammatory responses through reduction in serum levels of TNF-α and IL-1β and IAP in septic patients with intestinal dysfunction manifested syndrome of obstruction of the bowels Qi.

## 1 Introduction

Sepsis is a life-threatening organ dysfunction caused by a dysregulated host response to infection, according to the 3rd international consensus definitions for sepsis and septic shock.^[1]^ Despite significant recent advances in medical therapy and widespread adoption of international sepsis guidelines, sepsis is one of the leading causes of death in the intensive care unit (ICU).^[2,3]^ It is accepted that the pathogenesis of sepsis is closely related to an excessive production of proinflammatory cytokines such as tumor necrosis factor-α (TNF-α), interleukin-1 (IL-1) and IL-6 caused by bacterial toxins,^[4–6]^ and endotoxemia enhances production of endogenous cytokines, including TNF-α, IL-1, and IL-6.

Inflammatory cytokines injure intestinal mucosal barrier function, result in the translocation of bacteria, endotoxin, and various kinds of metabolites, as a result of the enterogenous bacteremia, eventually lead to sepsis and multiple organ dysfunction syndrome (MODS). Hence, the gut has long been hypothesized to be “the motor” of critical illness and specific sepsis, driving systemic inflammation through a number of disparate feedback and feedforward mechanisms.^[7]^

Normal intraabdominal pressure (IAP) in critically ill adults is regarded as 5 to 7 mm Hg. Intraabdominal hypertension (IAH) is defined by a sustained or repeatedly elevation of IAP above 12 mm Hg in adults and above 10 mm Hg in children and has 4 grades: grade I 12 to 15 mm Hg; grade II 16 to 20 mm Hg; grade III 21 to 25 mm Hg; and grade IV >25 mm Hg.^[8]^ IAH results in decrease in abdominal perfusion pressure, which is not only related with negative effects on splanchnic, respiratory, cardiovascular, renal, and neurological function but with mortality.^[9]^

Electro-acupuncture (EA), one of the therapeutic maneuvers in traditional Chinese medicine has been applied in clinics for thousands of years and has been evidenced to have a bidirectional neuron-endocrine-immune system regulating effect,^[10]^ so antagonize systemic inflammatory response without side effects. According to traditional Chinese medicine theory, the ST36 (Zusanli) and ST37 (Shangjuxu) acupuncture points are located at 3 and 6 cm, respectively, below the knee joint on the anterior aspect of the leg. In animal studies, Du et al found that EA at ST36 relieved system inflammation in a rat ischemia model through activating the cholinergic antiinflammatory pathway.^[11]^ EA at ST36 may reduce the severity of acute pancreatitis by inducing antiinflammatory effects and reducing the time to refeeding in patients with acute pancreatitis and EA at ST36 accelerate the recovery of gastrointestinal motility after colorectal surgery.^[12,13]^ Wu et al^[14]^ indicated that EA at ST36-ST37 improved the immunological function, decreased intestinal permeability,^[15]^ and recovered intestinal function in patients with sepsis.^[16]^ EA also shortened period of recovery of bowel sound, improved the intestinal function, reduced complications in postoperative patients, and decreased the urinary bladder pressure in patients with acute gastrointestinal injury.^[17,18]^

During the clinical treatment of sepsis, acupuncture and other traditional methods are applied to treat intestinal dysfunction of sepsis, and some effects have been obtained. However, the effects of EA at ST36-ST37 on inflammatory responses and IAP in septic patients with intestinal dysfunction manifested syndrome of obstruction of the bowels Qi remain unknown. Therefore, the aim of this study was to determine whether EA at ST36-ST37 could attenuate inflammatory responses and IAP in septic patients with intestinal dysfunction manifested syndrome of obstruction of the bowels Qi.

## 2 Materials and methods

The study was conducted in accordance with the guidelines of the Declaration of Helsinki^[19]^ and was approved by the Ethics Committee of Tongde Hospital of Zhejiang Province (approval no. [2015]051-018). The trial was prospectively registered in the Chinese Clinical Trial Registry (registration no. ChiCTR-IOR-17010910) on March 18th, 2017.

### 2.1 Settings and patients

A target sample of 82 patients, aged 18 to 80 years, sepsis-induced intestinal dysfunction manifested syndrome of obstruction of the bowels Qi, were recruited at Tongde Hospital of Zhejiang Province between March 2017 and November 2017. All the participants received treatment in the ICU of this hospital.

Sepsis was diagnosed according to the criteria outlined in the Surviving Sepsis Campaign: International Guidelines for Management of Sepsis and Septic Shock: 2016 published in the Critical Care Medicine,^[20]^ and intestinal dysfunction was diagnosed according to the criteria outlined in the gastrointestinal function in intensive care patients: terminology, definitions, and management,^[21]^ while the syndrome of obstruction of the bowels Qi pattern was diagnosed according to an expert consensus of diagnosis and treatment of integrated traditional Chinese and Western medicine on sepsis.^[22]^

All patients included in this study or their relatives consented to participation. They had at least a type of infection and were diagnosed to be sepsis. Patients had suffered from intestinal dysfunction and syndrome of obstruction of the bowels Qi. Patients with malignant tumor, pregnancy, fainting during acupuncture, infected sites of ST36, ST37, and inflammatory bowel disease were excluded from this study.

Every potential participant or relative was evaluated and informed about the procedures as well as the risks involved with participation in this study at the initial interview, and a full past medical history was taken. Candidates who went through the preliminary evaluation and signed consent underwent further examination and those who satisfied all the inclusion criteria were enrolled in the ICU. Baseline demographic data including current age, sex, acute physiology and chronic health evaluation (APACHE)-II, sepsis-related organ failure assessment (SOFA) score, portion of septic shock, sources of infection, and underlying diseases were collected.

### 2.2 Treatment protocol

As our experimental approach, we used a randomized controlled trial design to examine the effects of EA at “Zusanli” (ST36) and “Shangjuxu” (ST37) on IAP and inflammatory responses in septic patients with intestinal dysfunction manifested syndrome of obstruction of the bowels Qi. After completing the baseline evaluation, patients were randomly assigned to either a control group or an EA group in a 1:1 ratio using a table of random numbers generated in SPSS software (version 19.0, SPSS Inc., Chicago, IL). In control group, patients were given mechanical ventilation (MV) using a volume controlled mode with a tidal volume of 6 mL/kg of predicted body weight to attain acceptable blood gases and were provided with midazolam and fentanil for sedation and analgesia, respectively, if necessary. And extubation was undertaken when indicated clinically. Extubation was performed when there was no evidence of cardiovascularly unstable, had arterial oxygen tension (PaO_2_) >80 mm Hg on an inspired oxygen concentration (FiO_2_) <40%, and a positive end-expiratory pressure (PEEP) <5 cmH_2_O. According to the protocol of Rivers et al,^[23]^ patients with sepsis-induced tissue hypoperfusion were provided with adequate initial resuscitation. Norepinephrine (3–40 μg/min) was given to maintain mean arterial pressure (MAP) at levels >65 mm Hg. The need for red blood cell (RBC) transfusion was determined based on the patient's hematocrit (HCT) concentration. Regular regimens were used in enteral nutrition; glutamine (0.2–0.4 mg/kg/day) was provided intravenously in patients on parenteral nutrition.

In addition to therapies mentioned above, patients in EA group were also provided with EA at ST36-ST37.

### 2.3 Electro-acupuncture

Each patient in EA group received EA twice a day for 5 days. After conventional disinfection with iodine and alcohol, 0.30 × 40 mm needles (Hwato, Suzhou, China) were inserted bilaterally 20 to 25 mm beneath the skin at ST36 and ST37 with manual lift-thrust to elicit qi (a characteristic needing sensation perceived by the subject while the acupuncturist felt a needle grasp). Then the needles were connected to an EA stimulator (KWD-808I, Changzhou, China). Stimulation was performed using a continuous wave, a frequency of 4 Hz, and the intensity was adjusted to induce visible muscle twitching for the duration of the 20-min EA period.

### 2.4 Measurement

Serum levels of inflammatory factors (TNF-α and IL-1β) and levels of IAP were measured at baseline and posttreatment day 1, 3, and 7.

Blood samples were drawn at baseline and at 1, 3, 7 days. Blood samples were immediately placed at room temperature for 10 minutes and then centrifuged at 3500 rpm for 15 minutes (80–2 centrifuge; Changzhou Guoyu Instrument Manufacturing Co., Ltd. China) and stored in a freezer at −80 °C until they were ready for processing and analysis. Serum TNF-α and IL-1β levels were measured using enzyme-linked immunosorbent assay (ELISA) kits (R&D Systems, Minneapolis, MN) according to the manufacturer's instructions and the data were analyzed. All the samples were discarded after the analysis.

The revised closed system repeated measurement technique was used for the measurement of bladder pressure.^[24]^ In this technique, a ramp with 3 stopcocks is inserted in the drainage tubing connected to a Foley catheter. A standard infusion set is connected to a 1000 mL saline bag and attached to the first stopcock. A 60 mL syringe is connected to the 2nd stopcock and the 3rd stopcock is connected to a pressure transducer via rigid tubing (ABV300, Cemma Enterprise Co, Ltd). The system is flushed with saline to remove air, and the pressure transducer is zeroed at the symphysis pubis. To measure IAP, the bladder is completely emptied and the urinary drainage tubing is clamped distal to the ramp-device. The desired amount of 25 mL of saline is aspirated from the bag into the syringe and then instilled in the bladder. After the stopcocks are opened to the pressure transducer, IAP can be read from the bedside monitor. To confirm correct measurement, a rapid flush test, inspection of respiratory pressure variations, and an oscillation test were performed before every measurement. After the system was flushed, baseline IAP was measured without instilling extra volume. Then IAP measurements were performed with 25 mL. Each instillation was followed by a 1-minute equilibration period. Each patient underwent repeated measurement series. All measurements were performed by the same observer to limit interobserver variability.

### 2.5 Statistical analyses

PASS software (version 11; NCSS, LLC) was used to calculate sample size. Sample size was determined two-sample *t* test power analysis using preliminary data obtained in our ICU with the following assumptions: α of 0.05 (2-tailed), power of 80%, the difference in the mean of IAP on day 7 between patients in control and EA group of 1.5 mm Hg, and a standard deviation of 0.3 mm Hg. Therefore, we calculated that a sample size of 41 would have an 80% power of detecting a difference at a 0.05 level of significance.

The statistical analyses were performed by a researcher who is blinded to the allocation. Following the per protocol (PP) principle, all the data were analyzed using SPSS software (version 19.0, SPSS Inc, Chicago, IL) and the results were presented as mean ± standard deviation (SD) or number (%).

Distributions of the discrete variables were compared between the 2 groups with either the Chi-square test or Fisher exact tests. Two-sample *t* test was used to compare between the 2 groups and paired *t* test to compare continuous variables before and after treatment. Patient survival was analyzed with the use of the Kaplan–Meier method and compared between groups with the use of the log-rank test. All tests were 2-tailed and *P* < .05 was considered to be statistically significant.

## 3 Results

**Figure 1:**
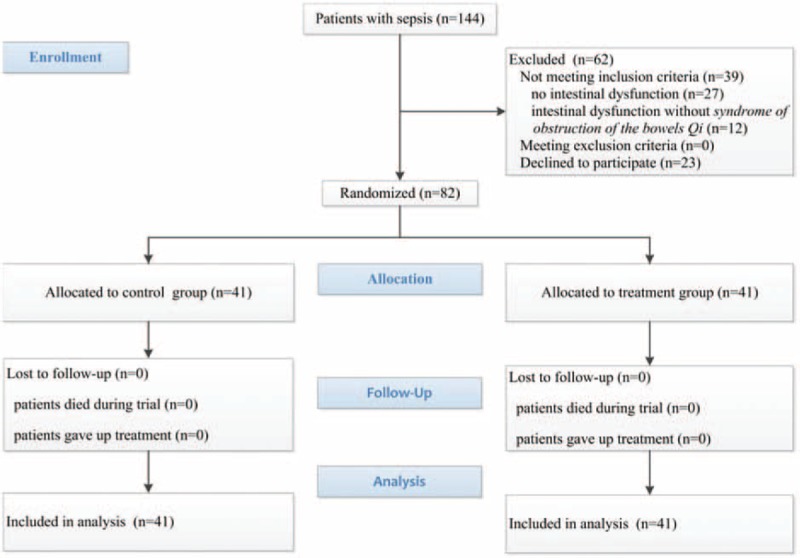
Diagram of the study.

During the period of recruitment from March 2017 to November 2017, 144 patients received a preliminary diagnosis of sepsis (Fig. 1). A total of 39 patients were excluded because they did not meet the formal diagnostic criteria for sepsis induced intestinal dysfunction manifested syndrome of obstruction of the bowels Qi, and 23 relatives of patients did not sign consent. Finally, 82 septic patients with intestinal dysfunction manifested syndrome of obstruction of the bowels Qi were enrolled in the study. None of patients lost to follow-up during period of the trial and therefore excluded from the primary statistical analysis. As a result, data from 82 participants were analyzed.

### 3.1 Baseline data

The demographic data were not significantly different between control group and EA group (*P* = .488, age and *P* = .508, male). The APACHE-II and SOFA scores at admission were not significantly different between control group and EA group: (*P* = .767, APACHE-II and *P* = .286, SOFA scores). The underlying diseases at admission were not significantly different between 2 groups (*P* = .733). There was no significant difference in the portion of septic shock (*P* = .206). The sources of infection which contributed to intestinal dysfunction in all patients included lung, abdomen, bloodstream, and urinary tract and none of them showed significant difference (*P* = .841) (Table 1).

**Table 1:**
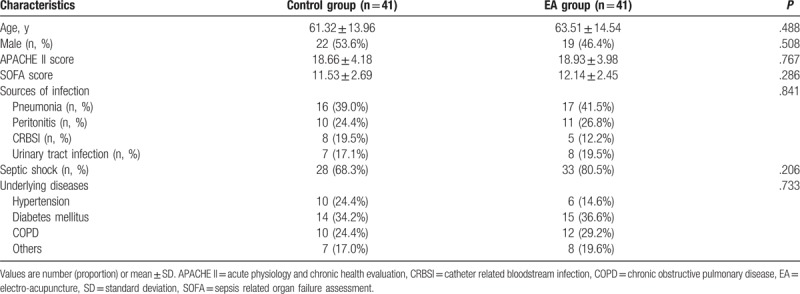
Baseline characteristics of participants.

### 3.2 Changes in plasma levels of inflammatory factors and IAP

There were no significant differences in levels of serum TNF-α, IL-1β, and IAP at baseline between 2 groups (*P* = .537, *P* = .291 and *P* = .120, respectively). After treatment, although the levels of serum TNF-α, IL-1β, and IAP decreased in 2 groups, TNF-α, IL-1β, and IAP decreased more significantly at day 1 day 3, and day 7 in EA group compared with control group (*P* < .005, *P* < .005, and *P* = .001, respectively, TNF-α; *P* = .032, *P* = .001, and *P* < .005, respectively, IL-1β; and *P* = .009, *P* < .005, and *P* < .005, respectively, IAP) (Table 2).

**Table 2:**
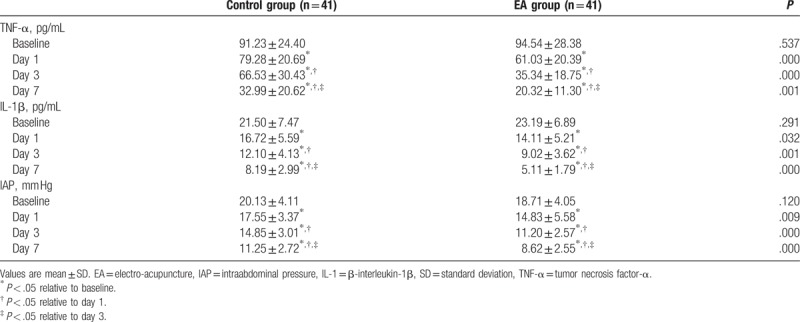
Serum inflammatory factors and IAP at different points in each group (
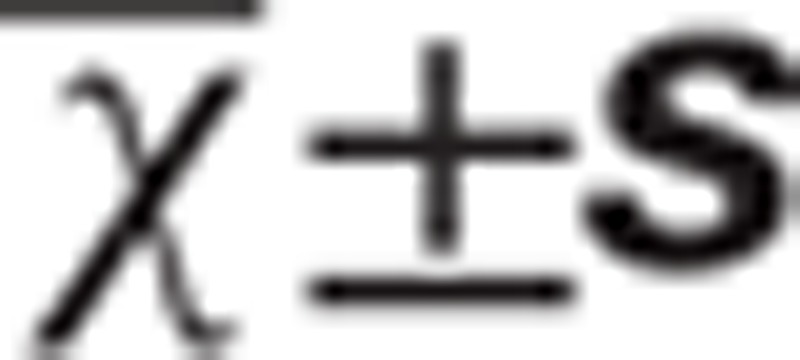
).

### 3.3 Prognosis

**Figure 2:**
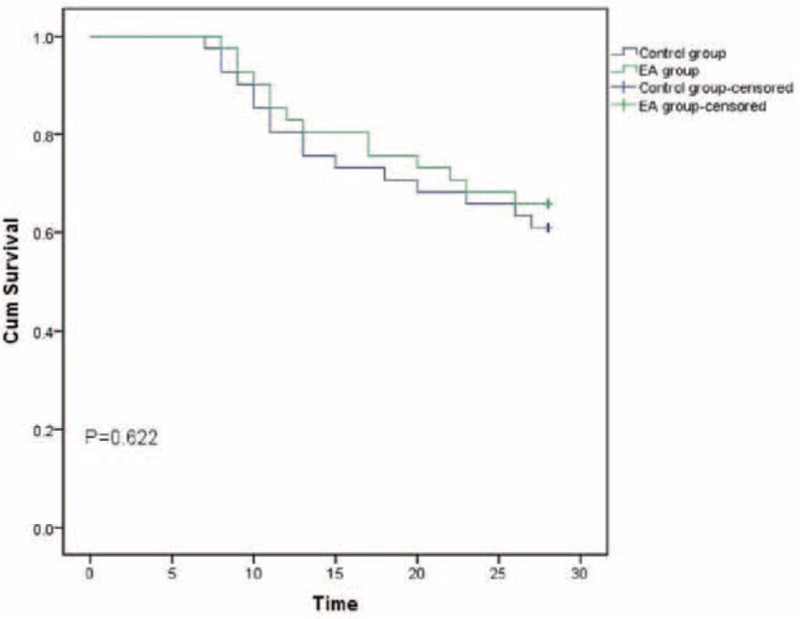
Kaplan–Meier plot of the probability of survival from randomization to day 28.

Compared to patients in control group, days on MV and length of stay in ICU were not shortened significantly in EA group (mean [SD]; 8.51[2.31] vs 7.80[2.03], *P* = .145, days on MV; mean [SD] 14.02[2.66] vs 13.66[2.81], *P* = .546, length of stay in ICU). And the overall mortality at day 28 did not show significant difference between 2 groups (number [%]; 16[39.0%] vs 14[34.2%], *P* = .647). A comparison of the 2 survival curves did not show significant difference (Fig. 2).

### 3.4 Safety of acupuncture

No adverse effects of acupuncture were documented during the study.

## 4 Discussion

The present study was designed to investigate the effects of EA at ST36-ST37 in septic patients with intestinal dysfunction manifested syndrome of obstruction of the bowels Qi. Comparing the EA group (n = 41), in which patients were expected to experience EA at ST36-ST37 by design, with the control group (n = 41), in which no EA at ST36-ST37 was expected, there were no significant differences in overall mortality at day 28 and survival curves and both groups showed similar days on MV and length of stay in ICU, suggesting the prognosis was not improved by EA at ST36-ST37. Superficial examination of our results may lead to a hasty conclusion that EA at ST36-ST37 is irrelevant for the protection in septic patients with intestinal dysfunction manifested syndrome of obstruction of the bowels Qi. However, we also evaluated the effects of EA at ST36-ST37 on serum cytokine levels (TNF-α and IL-1β) and IAP in septic patients with intestinal dysfunction manifested syndrome of obstruction of the bowels Qi. Our findings suggest that EA at ST36-ST37 not only attenuated inflammatory responses but also reduced IAP in septic patients with intestinal dysfunction manifested syndrome of obstruction of the bowels Qi.

The development of IAH has confirmed the concept that the activation of intestinal mucosal immune system induced by bacterial translocation and permeability changes may be the motor of MODS.^[25,26]^ Septic patients frequently suffer from syndrome of obstruction of the bowels Qi which are manifested abdominal distension, emesis, none of defecation and evacuation, and hypoactive bowel sounds. Intestinal evacuation will be dysfunction and flora, endotoxin, and bacteria in gut will move into blood when intestinal barrier is damaged by the decrease in blood circulation and ischemia reperfusion injury in intestines. Then multiinflammatory factors including TNF-α, IL-1, and IL-6 released into blood will trigger or aggravate systemic inflammatory response syndrome, which will lead to more seriously intestinal injury, enhance intestinal permeability and IAP, and induce IAH, abdominal compartment syndrome, and MODS.^[22,27]^ Therefore, to protect intestines and reduce the inflammatory response and IAP in septic patients, it is necessary to blockade or decrease the injury in intestines and production of proinflammatory mediators. In our study, EA at ST36-ST37 not only attenuated inflammatory responses but also reduced IAP in septic patients with intestinal dysfunction manifested syndrome of obstruction of the bowels Qi.

ST36 and ST37 are the points of the Stomach Meridian of Foot-Yangming, ST36 is the confluent point of the Stomach Meridian of Foot-Yangming, and ST37 is the lower confluent point of the Large Intestine Meridian of Hand-Yangming. And their principle therapeutic indications are for diseases of digestive system, by exerting the effects of dredging the excretory organs and immuno-regulation. Wu et al^[14]^ indicated that EA at points of the Stomach Meridian of Foot-Yangming could cut down the plasma levels of TNF-α inhibit the progression of inflammatory reaction in patients with sepsis. Wang et al^[17]^ showed that EA at ST36-ST37 could shorten recovery time of bowel sound, accelerate defection, and facilitate recovery of gastrointestinal function in patients suffered abdominal surgical procedures. In addition, EA at ST36-ST37 could relieve constipation and expel toxin through dredging meridian, regulating pure and turbid of the spleen and stomach, up and down of Qi movement, coordinating yin yang, and strengthening body resistance and fostering foundation of life.^[28]^ Yu et al^[18]^ found that EA at ST36-ST37 contributed to gastrointestinal motility recovery and decreased the urinary bladder pressure in the critically ill patients. However, the mechanism of antiinflammatory and reduction of IAP in septic patients is still unclear. Borvikova et al reported that “cholinergic anti-inflammatory pathway” could antagonize proinflammatory factors directly and alleviate the lethal effect of endotoxin and neuro-transmitter-acetylcholine (ACh) regulated systemic inflammation.^[29]^ Hence, the afferent somatic messages from ST36-ST37 by EA could be transmitted to nucleus of solitary tract, which regulate systemic inflammation, affect the motor activity of the stomach and gut and electrical release by efferent vagus nerve.^[30]^ The antiinflammatory and reduction in IAP induced by EA at ST36-37 maybe attribute to release of Ach by the vagus nerve as a result of transmission of message to vagus nuclei consequent on EA of ST36-ST37 acupoints and the bilateral division of vagus nerve. The mechanism of EA is very complicated and unclear, however, activation of cholinergic antiinflammatory pathway may be considered to be one of the main mechanisms of EA at ST36-ST37 in exerting the effect of attenuating inflammatory responses and IAP in septic patients with intestinal dysfunction manifested syndrome of obstruction of the bowels Qi.

In a previous study, EA for 30 minutes per day for 7 days at bilateral points ST36, Hegu (LI4), Zhigou (TE6), ST37, and Taichong (LR3) reduced the severity of acute pancreatitis (AP) by inducing antiinflammatory effects and reducing the time to refeeding.^[12]^ In addition, Li et al found that EA at ST36, LI4, Waiguan (TE5), Jinme (BL63), LR3, Qiuxu (GB40), Tianzhu (BL10), Fengchi (GB20), Cuanzhu (BL2), and Yuyao (EX-HN4) on the side with the craniotomy reduced immune suppression in patients undergoing supratentorial craniotomy.^[31]^ Although the antiinflammatory effects were induced and the immune suppression were reduced by EA at acupoints mentioned above, these acupoints they selected might too complicated to be implemented in critically ill patients. Preoperative EA at neiguan (PC6), ST36, and ST37 might be useful for preventing postoperative gastrointestinal dysfunction, thereby improving gastrointestinal function recovery.^[32]^ In our study, ST36-ST37 were only selected and we found that EA at ST36-ST37 not only attenuated inflammatory responses but also reduced IAP. Therefore, EA at ST36-ST37 is convenient, effective, and feasible in ICU.

### 4.1 Limitations

Although EA at ST36-ST37 was convenient and attenuated Serum levels of TNF-α and IL-1β and levels of IAP in our study, the prognosis was not improved by EA at ST36-ST37 in septic patients with intestinal dysfunction manifested syndrome of obstruction of the bowels Qi. We thought that the improvement in inflammatory response and IAP is still inadequate to affect the progress of pathogenetic progression and inverse the prognosis in these patients.

### 4.2 Future directions

This study was a small sample size and single-center trial and could not represent results for the general population completely. So, further investigations are required to refine our technique, expand sample size.

## 5 Conclusions

In conclusion, EA at ST36-ST37 attenuated inflammatory responses through reduction in serum levels of TNF-α and IL-1β levels of IAP in septic patients with intestinal dysfunction manifested syndrome of obstruction of the bowels Qi.

## Acknowledgments

The authors thank Grants No 2016ZA015 given by Zhejiang Provincial Administration of Traditional Chinese Medicine, China for the support.

## Author Contributions

All authors discussed the research process, results, and implications and commented on the manuscript.

Created and designed this study: Jian-biao Meng, Yan-na Jiao, Zhi-zhen Lai, Geng Zhang, Ma-hong Hu.

Collected and analyzed the data: Jian-biao Meng, Yan-na Jiao, Zhi-zhen Lai, Chun-lian Ji.

Contributed to the preparation and editing of the manuscript: Jian-biao Meng, Yan-na Jiao, Zhi-zhen Lai, Ma-hong Hu.

**Conceptualization:** Zhi-zhen Lai, Geng Zhang, Ma-hong Hu.

**Data curation:** Jian-biao Meng, Xiu-juan Xu, Chun-lian Ji.

**Formal analysis:** Xiu-juan Xu.

**Funding acquisition:** Zhi-zhen Lai.

**Investigation:** Jian-biao Meng, Yan-na Jiao, Xiu-juan Xu, Zhi-zhen Lai, Chun-lian Ji.

**Methodology:** Jian-biao Meng, Yan-na Jiao, Zhi-zhen Lai, Ma-hong Hu.

**Visualization:** Geng Zhang, Ma-hong Hu.

**Writing – original draft:** Jian-biao Meng.

**Writing – review & editing:** Jian-biao Meng, Yan-na Jiao, Zhi-zhen Lai, Geng Zhang, Chun-lian Ji, Ma-hong Hu.
